# Adjunctive Manual Thrombus Aspiration during ST-Segment Elevation Myocardial Infarction: A Meta-Analysis of Randomized Controlled Trials

**DOI:** 10.1371/journal.pone.0113481

**Published:** 2014-11-18

**Authors:** Song-Bai Deng, Jing Wang, Jun Xiao, Ling Wu, Xiao-Dong Jing, Yu-Ling Yan, Jian-Lin Du, Ya-Jie Liu, Qiang She

**Affiliations:** 1 Department of Cardiology, The Second Affiliated Hospital of Chongqing Medical University, Chongqing, China; 2 Department of Cardiology, The Medical Emergency Center of Chongqing, Chongqing, China; Azienda Ospedaliero-Universitaria Careggi, Italy

## Abstract

**Objective:**

The aim of this study was to synthesize evidence by examining the effects of manual thrombus aspiration on clinical outcomes in patients with ST-segment elevation myocardial infarction (STEMI).

**Methods and Results:**

A total of 26 randomized controlled trials (RCTs), enrolling 11,780 patients, with 5,869 patients randomized to manual thrombus aspiration and 5,911 patients randomized to conventional percutaneous coronary intervention (PCI), were included in the meta-analysis. Separate clinical outcome analyses were based on different follow-up periods. There were no statistically reductions in the incidences of mortality (risk ratio [RR], 0.86 [95% confidence interval [CI]: 0.73 to 1.02]), reinfarction (RR, 0.62 [CI, 0.31 to 1.32]) or target vessel revascularization (RR, 0.89 [CI, 0.75 to 1.05]) in the manual thrombus aspiration arm at 12 to 24 months of follow-up. The composite major adverse cardiac events (MACEs) outcomes were significantly lower in the manual thrombus aspiration arm over the long-term follow-up (RR, 0.76 [CI, 0.63 to 0.91]). A lower incidence of reinfarction was observed in the hospital to 30 days (RR, 0.59 [CI, 0.37 to 0.92]).

**Conclusion:**

The present meta-analysis suggested that there was no evidence that using manual thrombus aspiration in patients with STEMI could provide distinct benefits in long-term clinical outcomes.

## Introduction

Thrombus aspiration for ST-segment elevation myocardial infarction (STEMI) has been utilized for a long time and has received a level IIA endorsement according to the U.S. guidelines [1]. In the recent years, there has been increasing interest in manual thrombectomy devices, and the evidence to date has suggested that manual thrombus aspiration, but not mechanical aspiration, is beneficial in reducing major adverse cardiac events (MACEs), including mortality, compared with conventional percutaneous coronary intervention (PCI) alone [2]–[6]. In the largest randomized trial to date, the TASTE (Thrombus Aspiration in ST-Elevation myocardial infarction in Scandinavia) study suggested that routine manual thrombus aspiration before PCI provided no significant benefit to mortality over PCI alone in patients with STEMI at 30 days and 1 year of follow-up, settling the debate over the benefits of using manual thrombus aspiration in this setting [7], [8]. Recently, thrombectomy was downgraded in the ESC/EACTS revascularization guidelines from a class IIa level of evidence B recommendation to a class IIb level of evidence A recommendation [9]. Despite two well-done updated meta-analyses recently performed on this topic by Kumbhani DJ et al [5], [6], controversy exists regarding the combination of outcome effects over different follow-up durations. Because additional studies and prolonged follow-ups of earlier trials have now been reported, we performed an updated meta-analysis of the reperfusion markers of STEMI patients undergoing PCI with manual thrombus aspiration devices, and we performed separate analyses of clinical outcomes based on different follow-up periods. Because the Rescue (Boston Scientific) and TVAC (Thrombus Vacuum Aspiration Catheter, Nipro) catheters, attached to aspiration pumps for vacuum creation, were defined inconsistently as manual or mechanical thrombectomy in previous published meta-analyses [2]–[6], these two catheters were classified as special thrombectomy devices in the present meta-analysis.

## Methods

This study was performed in compliance with the quality of reporting for meta-analyses (PRISMA [Preferred Reporting Items for Systematic reviews and Meta-Analyses] statement) [10].

### Data Sources and Searches

We performed a computerized literature search of the PubMed, Web of Science, and Central databases for relevant articles published until September 2014, using the Medical Subject Heading and keyword search terms myocardial infarction, ST-segment elevation myocardial infarction, STEMI, thrombus aspiration, thrombectomy, Diver, Pronto, Export, Thrombuster, Eliminate, Rescure, TVAC, revascularization, percutaneous coronary intervention, angioplasty and PCI. No restrictions were applied to the publication period of the articles. This search was supplemented with citation tracking of relevant review articles and prior meta-analyses. Furthermore, conference proceedings from the American College of Cardiology, American Heart Association, European Society of Cardiology, EuroPCR scientific sessions and Transcatheter Cardiovascular Therapeutics were scanned. Only English-language studies were included.

### Study Selection

We selected studies in which patients with STEMI undergoing primary PCI or rescue PCI were randomly assigned either to manual thrombus aspiration followed by PCI or to PCI only. We only included studies that reported clinical outcome data and/or markers of post-procedure myocardial reperfusion. We excluded studies that performed thrombectomy only on saphenous vein grafts, studies that performed mechanical thrombectomy, studies of elective PCI and studies that compared one thrombectomy device to another.

### Data Extraction and Quality Assessment

The data were independently abstracted by two reviewers (Song-Bai Deng, Ling Wu). Agreement between the reviewers was evaluated by Kappa statistics. Disagreements were resolved through discussion, and a third reviewer (Qiang She) was involved to achieve a consensus when necessary. The bias of the included studies was assessed by the Cochrane group's *Cochrane Handbook for Systematic Reviews of Interventions* [11].

### Data Synthesis and Analysis

The primary clinical endpoint was all-cause mortality. The secondary endpoints were MACEs (composite of death, reinfarction, and target vessel revascularization), reinfarction, target vessel revascularization (TVR) and stent thrombosis. Angiographic and electrocardiographic outcomes that reflected post-procedure myocardial reperfusion included post-procedure myocardial blush grade (MBG) 3, thrombolysis and thrombin inhibition in myocardial infarction (TIMI) 3, and resolution of ST segment elevation (STR) >70%. We performed separate analyses of clinical outcomes based on different follow-up periods. The time frames were defined to reflect short-term (in hospital to 30 days), medium-term (6 to 9 months) and long-term (longer than or equal to 1 year) follow-ups, according to the different follow-up durations of the included studies. If manual and mechanical devices were both used in the same study, only data pertaining to manual aspiration thrombectomy were extracted. A subanalysis of the special thrombectomy devices (Rescue and TVAC) was performed. For all of the clinical outcomes, intention-to-treat analysis was utilized. The meta-analysis was performed using RevMan software, version 5.3 (Cochrane Collaboration). Summary risk ratios (RRs) and their corresponding 95% confidence intervals (CIs) were computed for each dichotomous outcome, using fixed or random effects models. For outcomes with significant heterogeneity (Chi^2^ p<0.05 or I^2^>50%), the random effects model is reported in the text and figures; for all of the other outcomes, the fixed effects models are reported. The random effects models were employed for sensitivity analysis when the fixed effects models produced positive results.

### Outcome Quality Assessment

We evaluated the level of evidence using the GRADE (Grades of Recommendation, Assessment, Development and Evaluation) approach [12]. The GRADEpro software version 3.6 was used. We obtained our assessment by judging the designs of the studies, the risk of bias, inconsistency, and imprecision.

## Results

### Eligible studies

**Figure 1:**
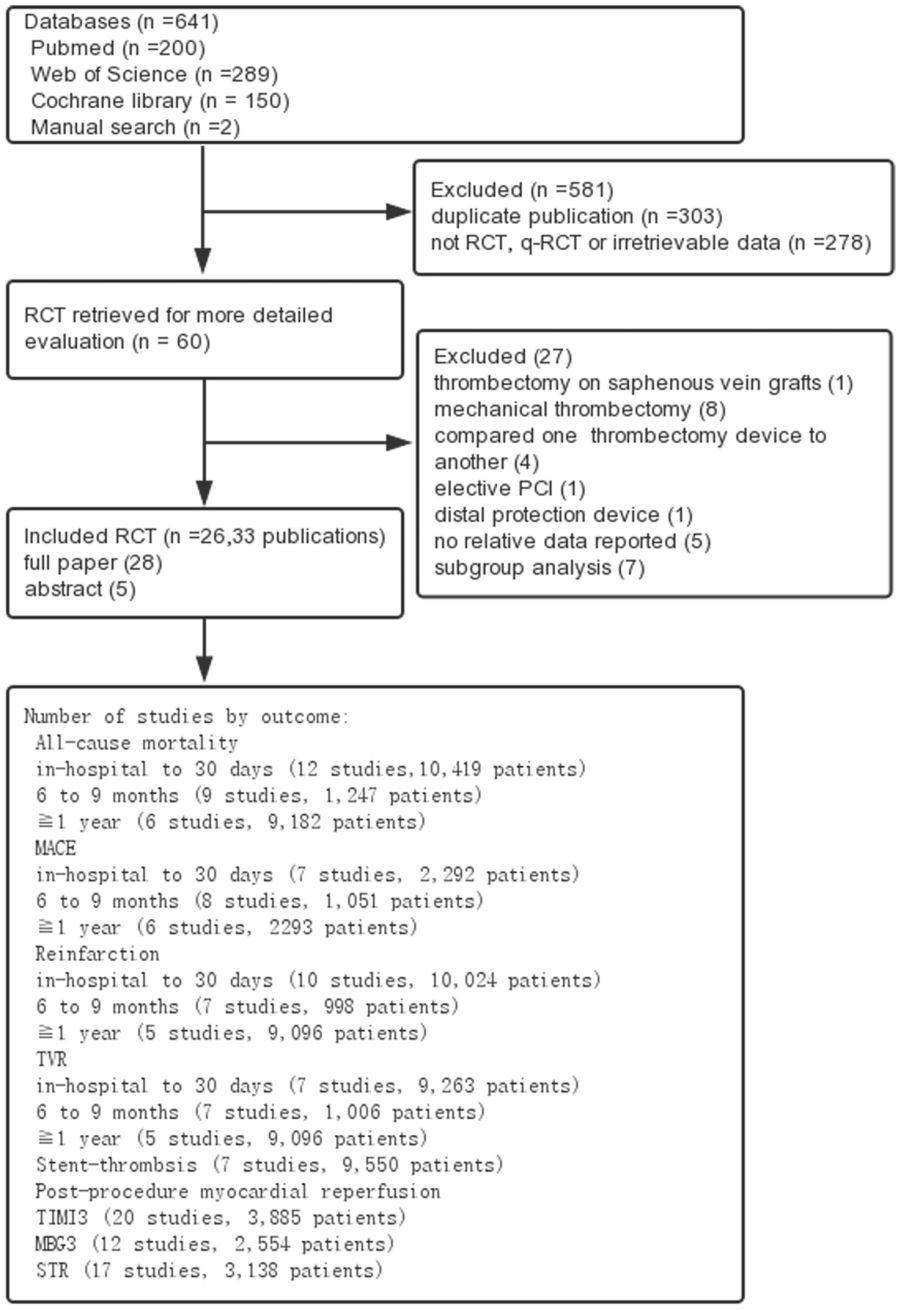
Flow diagram of the systematic overview process.

The initial search obtained 641 potentially relevant publications. After reading the abstracts and the full texts, 26 RCTs were finally included, enrolling 11,780 patients, with 5,869 patients randomized to manual thrombus aspiration and 5,911 patients randomized to conventional PCI (**Figure 1**). The follow-up periods varied between in-hospital and 5 years. Twelvetrials presented short-term follow-up results (in-hospital to 30 days) [9], [13]–[22], 8 trials presented medium-term follow-up results (6 to 8 months) [23], [29], and 2 trials presented long-term follow-up results (12 months) [30], [31]. In addition, 5trials presented different follow-up periods: the TAPAS (The Thrombus Aspiration during Percutaneous Coronary Intervention in Acute Myocardial Infarction Study) [32], [33] and INFUSE-AMI (Infuse–Acute Myocardial Infarction; An Optical Frequency Domain Imaging Study) [34], [35] studies followed up from 30 days and then to up to 12 months, TASTE followed up from 30 days to 1 year [7], [8], EXPIRA (The Thrombectomy With Export Catheter in Infarct-Related Artery During Primary Percutaneous Coronary Intervention Prospective, Randomized Trial) from 9 months and up to 24 months [36], [37] and VAMPIRE (VAcuuM asPIration thrombus Removal trial) from 1 month to 5 years [38]–[41]. Kappa statistics showed good agreement between the reviewers in the selection and data extraction (Kappa = 0.85). Among 24 included studies, most of them did not explicitly describe random sequence generation or allocation, and few presented attrition or reporting bias. Because the participants and personnel could not be blinded to the trials, the vast majority of studies were only blinded to outcome assessment. The reviewers' judgments about each risk of bias item are presented in **Figure S1**. The baseline characteristics of the included patients are listed in **Table 1**.

**Table 1:** Baseline characteristics of trials included in the meta-analysis.

			Manual Thrombus Aspiration/Conventional Primary PCI						
Study/Ref	Design	Device	n	Mean Age, Yrs	Male,%	Baseline TIMI 0/1, %	Ischaemia time,h	GP IIb/IIIa inhibitor, %	Follow-Up time, months
TASTE [7], [8]	multicenter	Eliminate/Pronto/Export	3621/3623	66.5/65.9	75.1/74.6	77.9/77.6	3.1/3.0†	15.4/17.4	1 m
REMEDIA [13]	single-center	Diver CE	50/49	61/60	90.0/77.6	86.0/89.8	4.6/5.0	68.0/63.3	1 m
Noel B et al [14]	single-center	Export	24/26	58/62	NA	NA	5.2/4.2	NA	H
DEAR-MI [15]	single-center	Pronto	74/74	57.3/58.9	84/76	81/73	3.4/3.3	100/100	H
EXPORT [16]	multicenter	Export	120/129	59.2/61.2	80.8/81.4	99.2/100	6.0/5.1	57.1/73.7	1 m
Lipiecki et al [17]	single-center	Export	20/24	59/59	60/75	100/96	7.1/7.4	5/12	H
Ciszewski et al [18]	single-center	Rescue/Diver CE	67/70	64.2/64.1	72/71	90/91	5.6/5.6	84/80	H
TROFI [19]	multicenter	Eliminate	71/70	61.1/60.9	75.7/69.1	48/46.4	NA	47.8/62.8	H
Dudek et al [20]	single-center	Rescue	40/32	57/59	NA	79/66	4.3/3.9	0/0	H
Kaltoft et al [21]	single-center	Rescue	108/107	65/11	76/80	68/69	4.0/3.5†	96/93	1 m
NONSOP [22]	multicenter	Rescue	129/129	64/65.9	79.8/79.8	NA	NA	NA	H
De Luca et al [23]	single-center	Diver CE	38/38	66.7/64.6	71.0/55.3	100/100	7.2/7.6	100/100	6 m
Chao et al [24]	single-center	Export	37/37	60/62	83.8/81.1	NA	5.6/5.9	19/32	6 m
Liistro et al [25]	single-center	Export	55/56	64/65	78/77	69/76	3.2/3.5	100/100	6 m
PIHRATE [26]	multicenter	Diver CE	100/96	60.8/58.8	80/81.7	96.7/97.9	NA	8/10.5	6 m
Bulum et al [27]	single-center	Export	30/30	54.3/58.5	83.3/73.3	NA	3.9/4.9	96.7/83.3	6 m
ITTI [28]	multicenter	Thrombuster II	52/48	60.5/56.5	90/81	83/92	2/8	54/52	6 m
Woo SI et al [29]	single-center	Export	33/30	55/53	84.8/100	78.8/83.3	4.4/4.7	0/0	6 m
MUSTELA [30]	multicenter	Export	50/104	62/63*	88.4/76*	91.3/77.9*	3.8/3.5*	100/100	12 m
Sim et al [31]	single-center	Thrombuster II	43/43	63/60	67.4/69.8	76.8/76.8	4.1/3.1†	30.2/46.5	12 m
TAPAS [32], [33]	single-center	Export	535/536	63/63	67.9/73.1	54.8/59.5	3.2/3.1	93.4/89.9	1 m,12 m
INFUSE-AMI [34], [35]	multicenter	Export	229/223	61/59	73.8/74	73.4/70	2.4/2.7	50.7/50.2	1 m,12 m
EXPIRA [36], [37]	single-center	Export	88/87	66.7/64.6	57/48	100/100	6.2/6.1	100/100	9 m,24 m
VAMPIRE [38]–[41]	multicenter	TAVC	180/175	63.2/63.5	80.6/77.7	74.6/75.3	6.3/7.1	0/0	1 m,8 m,5years
Hamza et al [42]	single-center	Diver CE	25/25	53.7/56.2	88/96	NA	5.91‡	100/100	H
Shehata et al [43]	single-center	Export	50/50	60.3/59.4	62/66	NA	NA	100/100	8 m

REMEDIA = The Randomized Evaluation of the Effect of Mechanical Reduction of Distal Embolization by Thrombus-Aspiration in Primary and Rescue Angioplasty Trial; TASTE = Thrombus Aspiration in ST-Elevation myocardial infarction in Scandinavia; DEAR-MI = The Dethrombosis to Enhance Acute Reperfusion in Myocardial Infarction Study; EXPORT = A Multicentre Randomized Controlled Trial of The EXPORT Aspiration Catheter; TROFI = Randomized Study to Assess the Effect of ThRombus Aspiration on Flow Area in Patients with ST-Elevation Myocardial Infarction; NONSOP = Intracoronary Aspiration before coronary Stenting in Patients with Acute Myocardial Infarction; PIHRATE = the Polish-Italian-Hungarian RAndomized ThrombEctomy Trial; ITTI = The Initial Thrombosuction and Tirofiban Infusion trial; MUSTELA = MUltidevice Thrombectomy in Acute ST-Segment Elevation Acute Myocardial Infarction Trial; TAPAS = The Thrombus Aspiration during Percutaneous Coronary Intervention in Acute Myocardial Infarction Study; INFUSE-AMI = Infuse–Acute Myocardial Infarction; An Optical Frequency Domain Imaging Study; EXPIRA = The Thrombectomy With Export Catheter in Infarct-Related Artery During Primary Percutaneous Coronary Intervention Prospective, Randomized Trial; VAMPIRE = VAcuuM asPIration thrombus Removal trial;

*Values for all thrombectomy (manual and mechanical) vs. conventional PCI alone;

† Median;

‡ mean ischaemia time of all patients;

NA: not available; H: In-hospital clinical outcomes.

### Mortality

**Figure 2:**
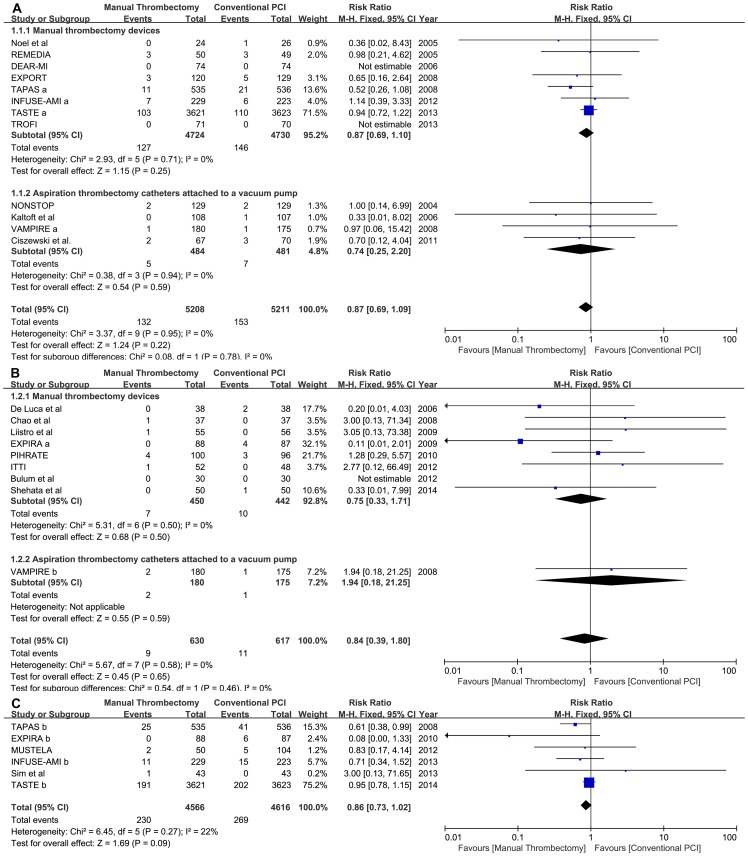
Forest plots for mortality in different follow-up periods. Footnote: A: short-term follow-up; B: medium-term follow-up; C: long-term follow-up; TAPAS a: 30-day of follow-up; TAPAS b: 12 months of follow-up; INFUSE-AMI a: 30-day of follow-up; INFUSE-AMI b: 12 months of follow-up; EXPIRA a: 6 months of follow-up; EXPIRA b: 24 months of follow-up; VAMPIRE a: 30-day of follow-up; VAMPIRE b: 8 months of follow-up. TASTE b, 1 year of follow-up.

There was no significant mortality benefit from in hospital to 30 days (2.53% thrombus aspiration vs. 2.94% conventional PCI; RR = 0.87, 95% CI 0.69 to 1.09, p = 0.22; p for heterogeneity [phet] = 0.95, I^2^ = 0%) or from 6 to 9 months of follow-up (1.43% vs. 1.78%; RR: 0.84, 95% CI: 0.39 to 1.80, p = 0.65; phet = 0.58, I^2^ = 0%). We observed a decreased trend toward mortality in manual thrombus aspiration with long-term follow-up (longer than or equal to 1 year), but this trend was not statistically significant (5.04% vs. 5.83%; RR: 0.86, 95% CI: 0.73 to 1.02, p = 0.09; phet = 0.27, I^2^ = 22%).A random-effects model yielded a similar result with long-term follow-up (RR: 0.80, 95% CI: 0.59 to 1.08, p = 0.14). Subgroup analysis of the different aspiration thrombectomy devices also showed similar results (**Figure 2**).

### MACEs

**Figure 3:**
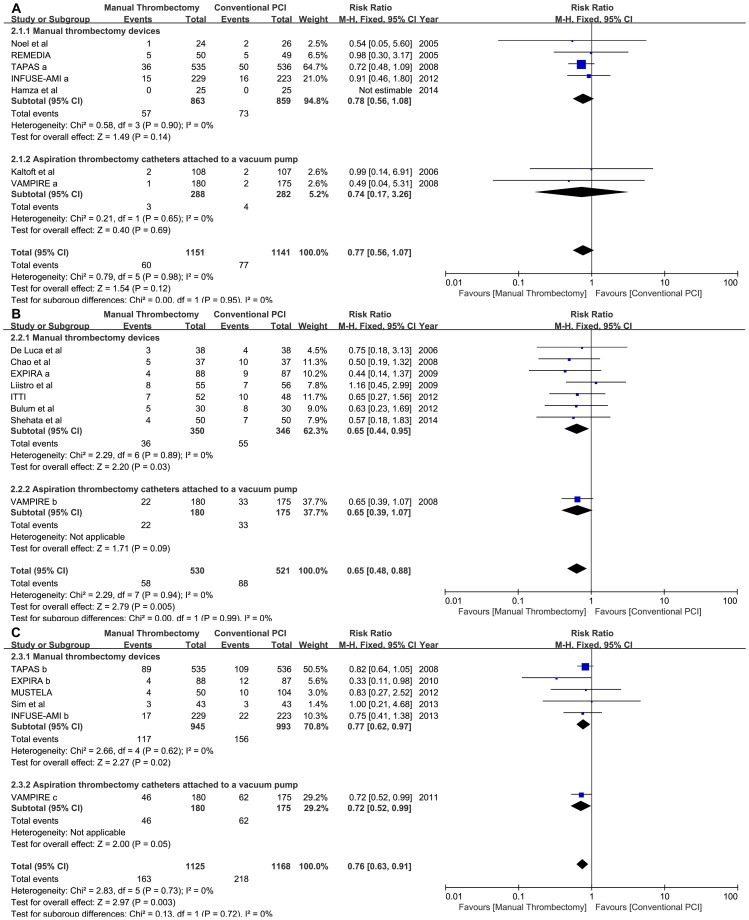
Forest plots for MACE in different follow-up periods. Footnote: A: short-term follow-up; B: medium-term follow-up; C: long-term follow-up; VAMPIRE c: 5 years of follow-up.

Because the MACEs was not pre-defined, the TASTE trail could not be included in the meta-analysis in terms of MACE outcomes. The composite MACE outcomes were significantly lower in the manual thrombus aspiration arm at 6 to 9 months (10.94% thrombus aspiration vs. 16.89% conventional PCI; RR: 0.65, 95% CI: 0.48 to 0.88, p = 0.005; phet = 0.94, I^2^ = 0%) and with long-term (14.49% vs. 18.66%; RR: 0.76, 95% CI: 0.63 to 0.91, p = 0.003; phet = 0.73, I^2^ = 0%) follow-up. A random-effects model yielded similar results (RR: 0.65, 95% CI: 0.48 to 0.88; RR: 0.76, 95% CI: 0.64 to 0.91, respectively). However, the composite MACEs were similar between the two arms from in hospital to 30 days of follow-up (4.34% vs. 6.75%; RR: 0.77, 95% CI: 0.56 to 1.07, p = 0.12; p_het_ = 0.98, I^2^ = 0%) (**Figure 3**).

### Reinfarction

**Figure 4:**
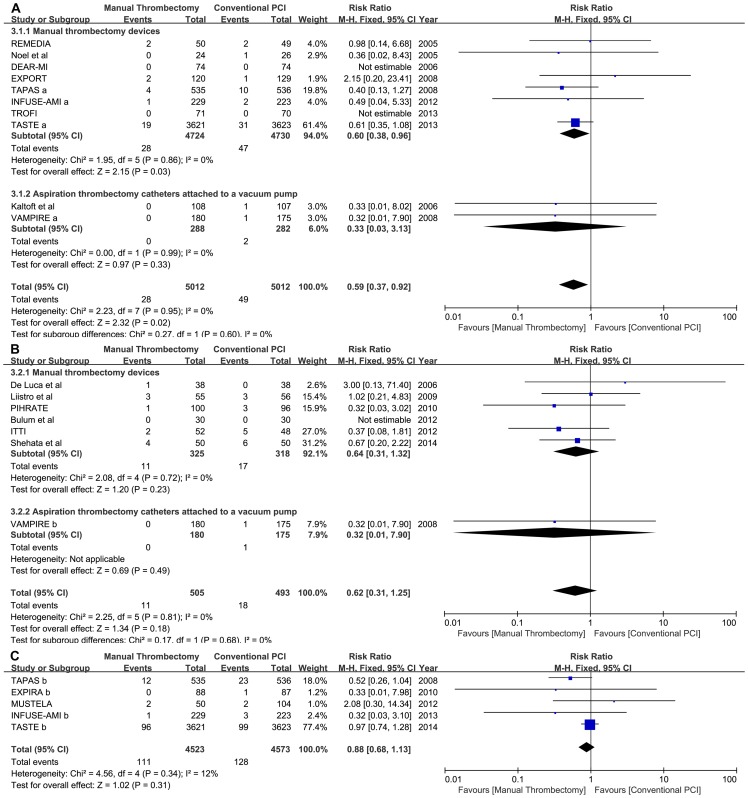
Forest plots for reinfarction in different follow-up periods. Footnote: A: short-term follow-up; B: medium-term follow-up; C: long-term follow-up.

The incidence of reinfarction was lower in the manual thrombus aspiration arm from in hospital to 30 days (0.56% thrombus aspiration vs. 0.98% conventional PCI; RR: 0.59, 95% CI: 0.37 to 0.92, p = 0.02; phet = 0.95, I^2^ = 0%) but not at 6 to 9 months (3.38% vs. 5.35%; RR:0.62, 95% CI: 0.31to1.25, p = 0.18; phet = 0.81, I^2^ = 0%) or with long-term (2.45% vs. 2.80%; RR:0.88, 95% CI: 0.68 to 1.13, p = 0.31; phet = 0.34, I^2^ = 12%) follow-up (**Figure 4**). A random-effects model yielded similar results from in hospital to 30 days of follow-up (RR: 0.59, 95% CI: 0.37 to 0.93, p = 0.02). Subgroup analysis suggested that the pure manual aspiration thrombectomy subgroup played a dominant role in the lower reinfarction incidence over short-term follow-up (**Figure 4**).

### Target vessel revascularization

**Figure 5:**
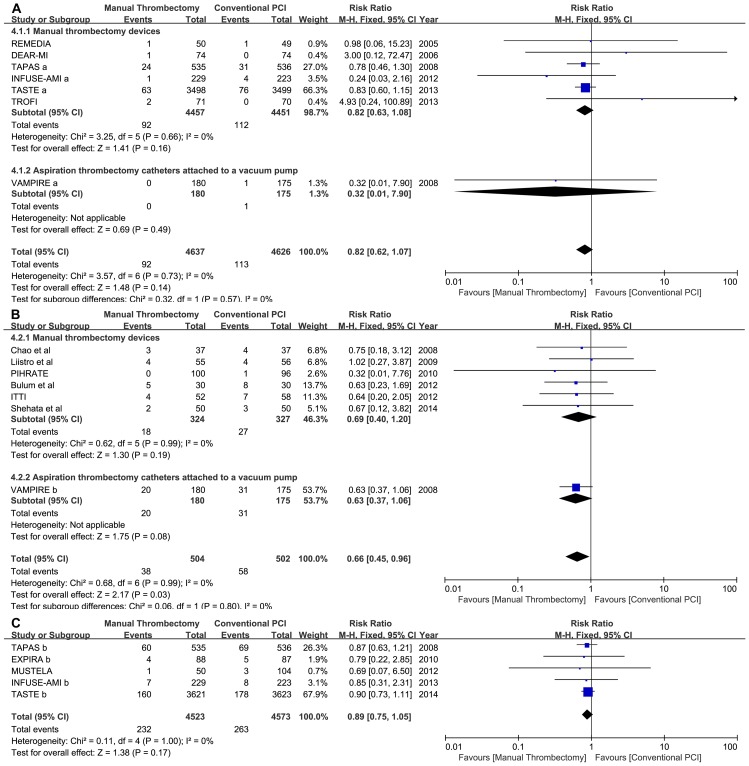
Forest plots for target vessel revascularization in different follow-up periods. Footnote: A: short-term follow-up; B: medium-term follow-up; C: long-term follow-up.

There were no differences between the two arms in the incidence of TVR from in hospital to 30 days (1.98% thrombus aspiration vs. 2.44% conventional PCI; RR: 0.82, 95% CI: 0.62 to 1.07, p = 0.14; p_het_ = 0.73, I^2^ = 0%) or with long-term (5.13% vs. 5.75%; RR: 0.89, 95% CI: 0.75 to 1.05, p = 0.17; p_het_ = 1.00, I^2^ = 0%) follow-up, but the incidence was significantly lower with manual thrombus aspiration from 6 to 9 months (7.54% vs. 11.55%; RR: 0.66, 95% CI: 0.45 to 0.96, p = 0.03; phet = 0.99, I^2^ = 0%) (**Figure 5**). However, subgroup analysis showed that the positive results were dominated by the VAMPIRE study, in which a special thrombectomy device was used (TVAC). When we excluded this study, there were no differences between the two arms (RR: 0.69, 95% CI: 0.40 to 1.20, p = 0.19).

### Stent thrombosis

**Figure 6:**
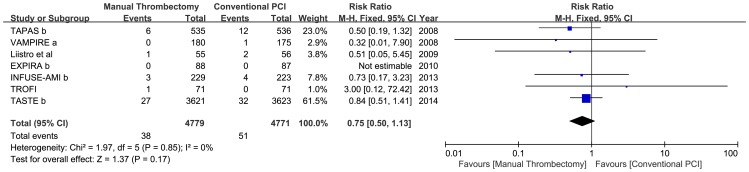
Forest plots for stent thrombosis.

Use of manual thrombus aspiration devices did not significantly reduce the incidence of total stent thrombosis (0.80% vs1.07%; RR: 0.75, 95% CI: 0.50 to 1.13, p = 0.17; p_het_ = 0.85, I^2^ = 0%). A random-effects model yielded similar results (RR: 0.75, 95% CI: 0.49 to 1.14, p = 0.18) (**Figure 6**).

### Markers of myocardial reperfusion

The use of manual thrombus aspiration devices was associated with significantly higher rates of post-procedure MBG 3 (RR: 1.43, 95% CI: 1.19 to 1.71, p<0.001; p_het_<0.0001, I^2^ = 73%), TIMI 3 flow (RR: 1.05, 95% CI: 1.02 to 1.09, p<0.001; p_het_ = 0.03, I^2^ = 40%), and STR >70% (RR: 1.27, 95% CI: 1.12 to 1.45, p<0.001; p_het_<0.0001, I^2^ = 69%). There were still significant advantages of angiographic and electrocardiographic outcomes in the manual thrombus aspiration arm when individually excluding the included trials. Subgroup analysis showed that the benefits of TIMI3 and STR mainly derived from the pure manual thrombectomy devices subgroup but not from the Rescue and TAVC subgroup (see **Figures S2, S3 and S4**). There was no evidence of publication bias in the included studies in the meta-analysis of post-procedure TIMI 3 flow, as visually analyzed with a funnel plot (see **Figure S5**).

### GRADE profile evidence

The GRADE system evidence for each outcome level and the reasons for upgrade and downgrade are shown in **Table 2**. According to the GRADE approach, the quality of evidence for the long-term follow-up of mortality, reinfarction and TVR was high, and the quality of evidence for stent thrombosis, long-term follow-up of MACEs and short-term follow-up of reinfarction was moderate.

**Table 2:** Summary of GRADE Evidence Profile of manual thrombectomy compared to conventional PCI for STEMI.

Outcomes	No of Participants (studies)	Quality of the evidence*	Relative effect	Anticipated absolute effects	
	Follow up	(GRADE)	(95% CI)	Risk with Conventional PCI	Risk difference with Manual thrombectomy (95% CI)
Long-term follow-up mortality	9182 (6 studies)	⊕⊕⊕⊕	RR 0.86	58 per 1000	8 fewer per 1000
	1 to 2 years	HIGH	(0.73 to 1.02)		(from 16 fewer to 1 more)
long-term follow-up MACEs	2293 (6 studies)	⊕⊕⊕⊝	RR 0.76	187 per 1000	45 fewer per 1000
	1 to 5 years	MODERATE1due to imprecision	(0.63 to 0.91)		(from 17 fewer to 69 fewer)
Short-term follow-up reinfarction	10024 (10 studies)	⊕⊕⊕⊝	RR 0.59	10 per 1000	4 fewer per 1000
	in-hospital to 30 days	MODERATE2due to imprecision	(0.37 to 0.92)		(from 1 fewer to 6 fewer)
long-term follow-up reinfarction	9096 (5 studies)	⊕⊕⊕⊕	RR 0.88	28 per 1000	3 fewer per 1000
	1 to 2 years	HIGH	(0.68 to 1.13)		(from 9 fewer to 4 more)
long-term follow-up TVR	9096 (5 studies)	⊕⊕⊕⊕	RR 0.89	58 per 1000	6 fewer per 1000
	1 to 2 years	HIGH	(0.75 to 1.15)		(from 14 fewer to 9 more)
Stent-thrombosis	9550 (7 studies)	⊕⊕⊕⊝	RR 0.75	11 per 1000	3 fewer per 1000
	in-hospital to 2 years	MODERATE3due to risk of bias	(0.5 to 1.13)		(from 5 fewer to 1 more)

*The basis for the assumed risk (e.g. the median control group risk across studies) is provided in footnotes. The corresponding risk (and its 95% confidence interval) is based on the assumed risk in the comparison group and the relative effect of the intervention (and its 95% CI). CI: Confidence interval; RR: Risk ratio; 1 No enough optimal information size; 2 Risk ratio <0.75; 3 Different definition of stent thrombosis outcome.

## Discussion

This meta-analysis performed separate analyses based on different follow-up periods to compare the clinical outcomes of manual thrombus aspiration with those of conventional PCI. The main findings of this meta-analysis were that the use of manual thrombus aspiration devices could significantly reduce the incidence of short-term reinfarction and long-term MACEs, but it did not result in lower rates of death, reinfarcion or TVR over long-term follow-up despite improved post-procedure myocardial reperfusion. These results were driven mainly by the TASTE trial. There were some differences from the well-done updated meta-analysis on this topic performed recently by Kumbhani DJ et al [5], [6]. First, we performed separate analyses of clinical outcomes based on different follow-up periods, and a subanalysis of the special thrombectomy devices (Rescue and TVAC) to avoid clinical heterogeneity as much as possible. Second, we included 6 additional studies and the results of the recent TASTE trial at the 1-year follow-up in this meta-analysis. Third, this meta-analysis presented fewer long-term clinical benefits of routine use of manual thrombus aspiration in patient with STEMI. Finally, we also assessed the level of evidence using the GRADE approach. The present meta-analysis showed that the composite MACE outcomes were significantly lower in the manual thrombus aspiration arm with long-term follow-up (RR: 0.76, 95% CI: 0.63 to 0.91). However, the number of included patients for this clinical outcome was far less than for the others because the TASTE trail could not be included due to MACEs not being pre-defined in this trail. In the TASTE trial, the composite incidence of death, rehospitalization for myocardial infarction, or stent thrombosis was 8.0% in the thrombus-aspiration group and 8.5% in the PCI-only group (hazard ratio, 0.94; 95% CI, 0.80 to 1.11; p = 0.48) [8]. Adding these factors together, we could not conclude that the use of manual thrombus aspiration devices could reduce the composite MACE outcomes.

In our meta-analysis, one trial warrants particular attention [7], [8]. The TASTE trial, a registry-based RCT, was a prospective, multicenter, controlled trial that randomly allocated 7,244 patients to undergo manual thrombus aspiration followed by PCI or PCI alone. The sample size was larger than those of all previous studies combined, and the power to detect differences at well-defined end points was much higher. The TASTE study did not show any significant differences in the primary outcome of all-cause mortality, and it showed non-significant trends toward less myocardial infarction and stent thrombosis at 30 days and 1 year of follow-up. Additionally, the outcome of thrombus aspiration in candidate subjects not enrolled in TASTE failed to show an advantage of this adjunct, although this meta-analysis found that adjunctive manual thrombosis aspiration significantly reduced the incidence of reinfarction at 30 days of follow-up. Based on the rate of reinfarction with short-term follow-up (0.56% vs.0.98%, separately), 238 patients needed to be treated to prevent 1 reinfarction event. Given that the price of an average aspiration catheter is approximately €250,the potential clinicoeconomic effectiveness of the use of routine manual thrombosis aspiration is low.

Different inclusion criteria and different manual aspiration thrombectomy devices were used in the various trials, and it is not surprising that there was significant statistical heterogeneity in the results of post-procedure myocardial reperfusion. For example, myocardial reperfusion was not improved and infarct size was not reduced by manual aspiration thrombectomy in the INFUSE-AMI trial of patients with large anterior STEMI [34]. Post-procedure myocardial reperfusion improvements were observed despite the inclusion of the recent INFUSE-AMI trial. A random effects model was employed in the meta-analysis, and there were still significant advantages of angiographic and electrocardiographic outcomes in the manual thrombus aspiration arm when individually excluding the included trials.

In the real world, the benefits of using manual thrombus aspiration in patients with STEMI are also controversial. One study found that the routine use of thrombus aspiration was associated with reduced 12-month mortality in a large real-world patient cohort [42]. These data supported the observed survival benefit in the TAPAS trial. However, another study showed that one-year mortality was similar in both groups in a real-world STEMI population [43]. Recently, thrombectomy was downgraded in the ESC/EACTS revascularization guidelines from a class IIa level of evidence B recommendation to a class IIb level of evidence A recommendation. What will change in the U.S. STEMI guidelines is unknown. These results might have caused uncertainty in the minds of some cardiologists regarding the utility of adjunctive thrombus aspiration for primary PCI patients. Thus, a subsequent analysis of the ongoing large-scale randomized trial (ClinicalTrials.gov number, NCT01149044) is imperative [44]. The TOTAL trial (A Randomized Trial of Routine Aspiration ThrOmbecTomy With PCI Versus PCI ALone in Patients With STEMI Undergoing Primary PCI) is an international, randomized, controlled, parallel-group study in which an estimated enrollment sample of 10,700 patients with STEMI will be allocated to manual aspiration thrombectomy with PCI or PCI alone, with follow-up of up to 180 days, and the primary end points are the first occurrence of cardiovascular death, recurrent myocardial infarction, cardiogenic shock, or new or worsening NYHA Class IV heart failure. In 2015 the results from the TOTAL trial are expected, and an updated meta-analysis including these data should be conducted. In clinical practice, thrombectomy catheters could be used to reduce thrombus burden by aspirating thrombi prior to stenting or balloon angiography; better reperfusion is predicted, and technically and procedurally using aspiration is also important. However, the use of thrombus aspiration during PCI in STEMI remains controversial

Our review had some limitations. First, this meta-analysis was not performed on individual patient data because complete data sets were not available. Second, only the TASTE trial was powered for mortality and the other clinical events reported, and the other included trials' sample sizes were small. Third, the number of screened subjects or percentages of included vs. candidate subjects in a number of included studies were not accounted for [13], [15], [20], [21], [24], [26], which is an important limitation that confounds how we should interpret our selection and outcome results.

## Conclusions

In summary, the present meta-analysis suggested that the use of manual thrombus aspiration devices could improve post-procedure myocardial reperfusion, but there was no evidence of a benefit in long-term clinical outcomes.

## Supporting Information

Figure S1 **Review authors' judgements about each risk of bias item presented as percentages across all included studies.**
(TIF)

Figure S2 **Forest plots for post-procedure MBG 3.** Footnote: TAPAS a: 30-day of follow-up; VAMPIRE a: 30-day of follow-up.
(TIF)

Figure S3 **Forest plots for post-procedure TIMI 3.** Footnote: TAPAS a: 30-day of follow-up; INFUSE-AMI a: 30-day of follow-up; VAMPIRE a: 30-day of follow-up.
(TIF)

Figure S4 **Forest plots for post-procedure STR.** Footnote: TAPAS a: 30-day of follow-up; INFUSE-AMI a: 30-day of follow-up; EXPIRA a: 6 months of follow-up; VAMPIRE a: 30-day of follow-up.
(TIF)

Figure S5 **Funnel plot of the included studies in meta-analysis of post-procedure TIMI 3 flow.** Footnote: The inverted and symmetrical funnel aspect can be observed for the assessed end points, with 95% of the studies lying within the confidence limit lines. This suggests that publication bias is not present among the included studies for the meta-analysis.
(TIF)

Checklist S1 PRISMA checklist.
(DOC)
