# Supplementary figures and images for: Adjunctive Manual Thrombus Aspiration during ST-Segment Elevation Myocardial Infarction: A Meta-Analysis of Randomized Controlled Trials
Source: PLoS One. 2014 Nov 18;9(11):e113481. doi: 10.1371/journal.pone.0113481 (PMC4236171; doi:10.1371/journal.pone.0113481)

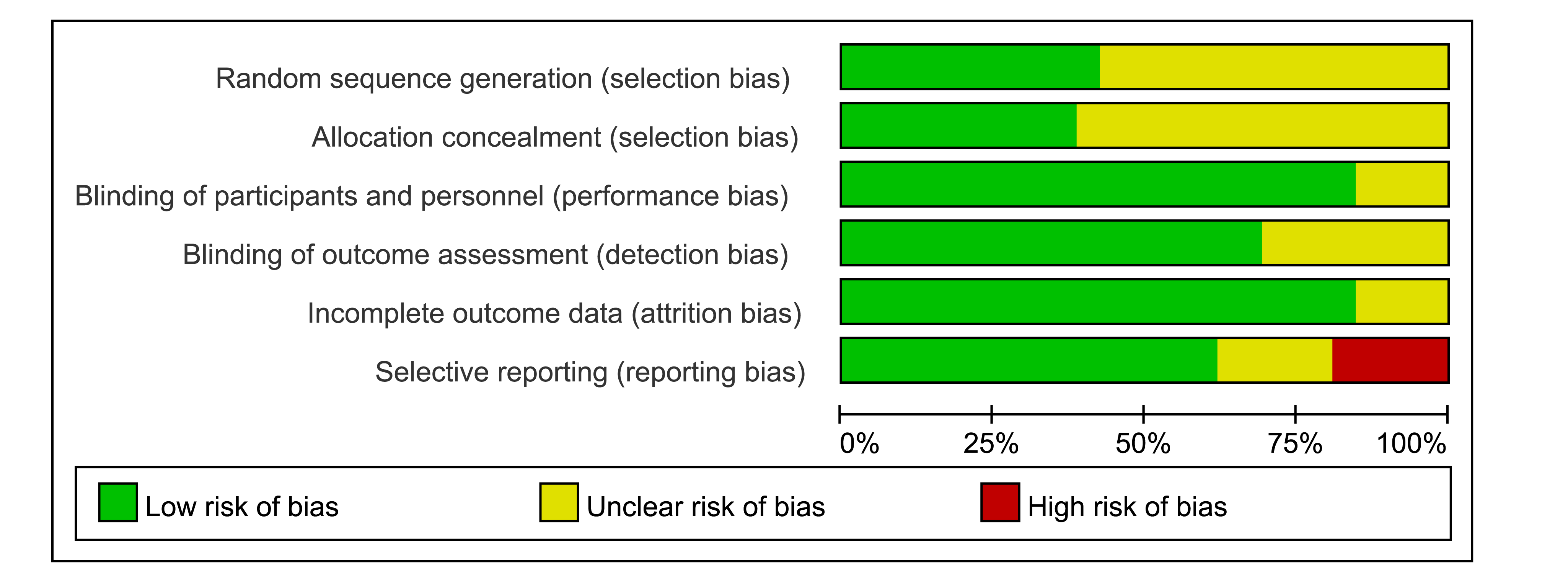

Supplement: Figure S1 — Review authors' judgements about each risk of bias item presented as percentages across all included studies. (TIF) [file pone.0113481.s001.tif]

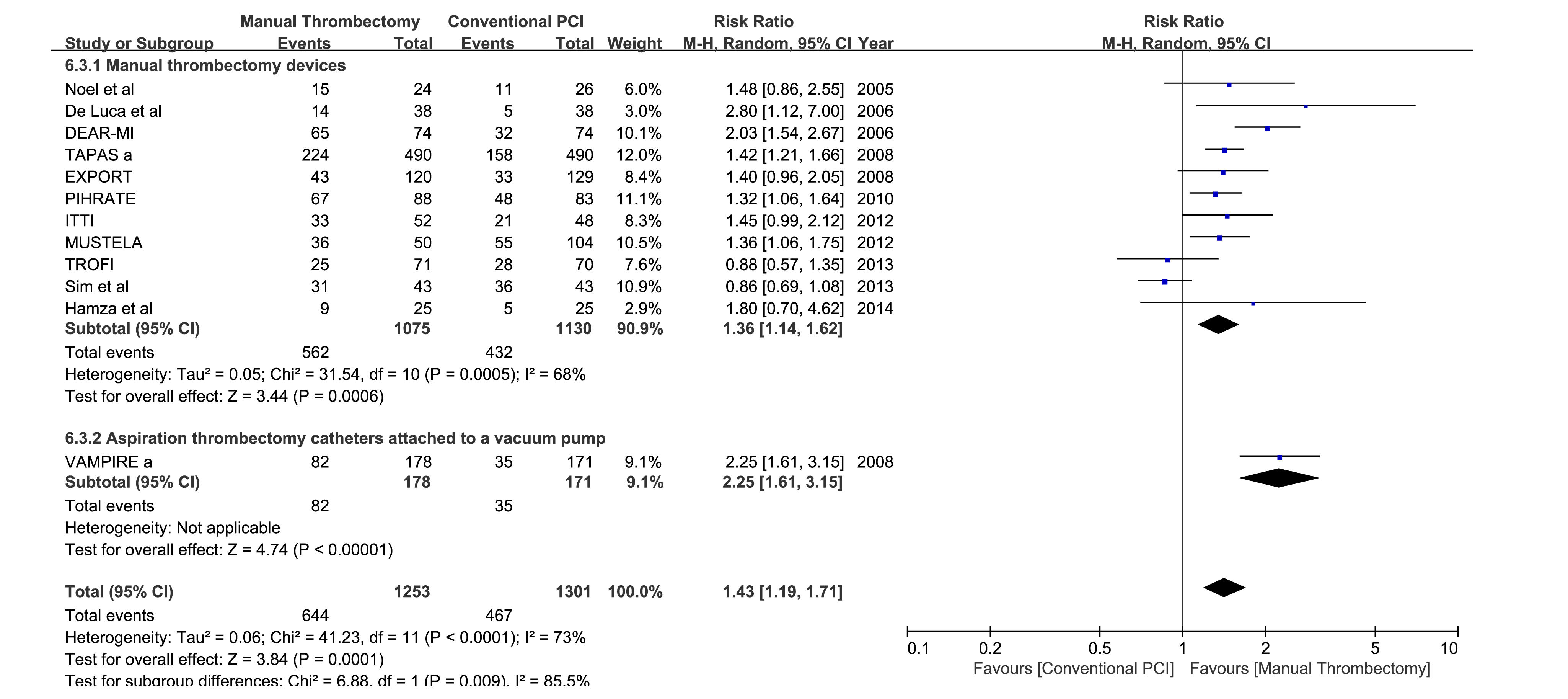

Supplement: Figure S2 — Forest plots for post-procedure MBG 3. Footnote: TAPAS a: 30-day of follow-up; VAMPIRE a: 30-day of follow-up. (TIF) [file pone.0113481.s002.tif]

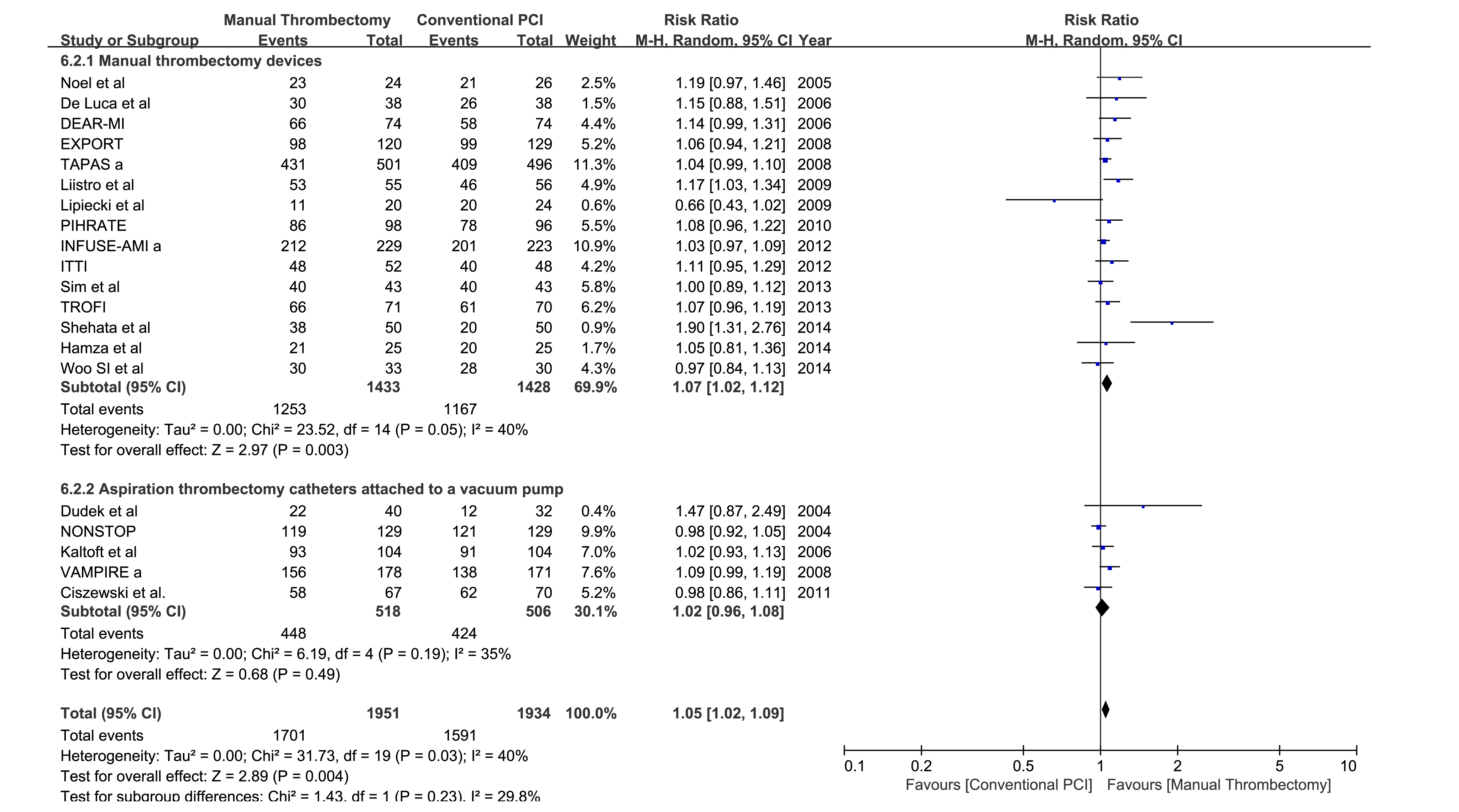

Supplement: Figure S3 — Forest plots for post-procedure TIMI 3. Footnote: TAPAS a: 30-day of follow-up; INFUSE-AMI a: 30-day of follow-up; VAMPIRE a: 30-day of follow-up. (TIF) [file pone.0113481.s003.tif]

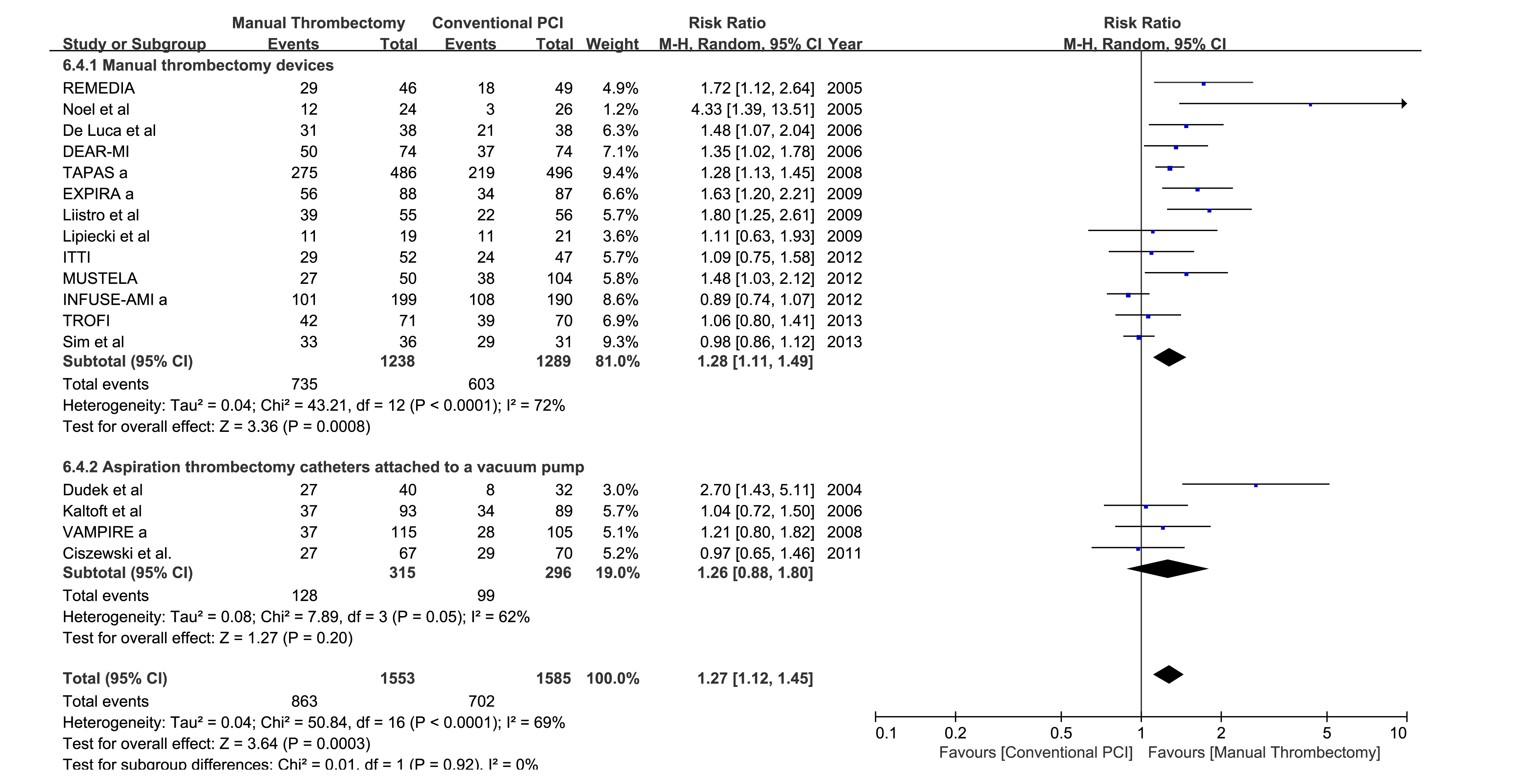

Supplement: Figure S4 — Forest plots for post-procedure STR. Footnote: TAPAS a: 30-day of follow-up; INFUSE-AMI a: 30-day of follow-up; EXPIRA a: 6 months of follow-up; VAMPIRE a: 30-day of follow-up. (TIF) [file pone.0113481.s004.tif]

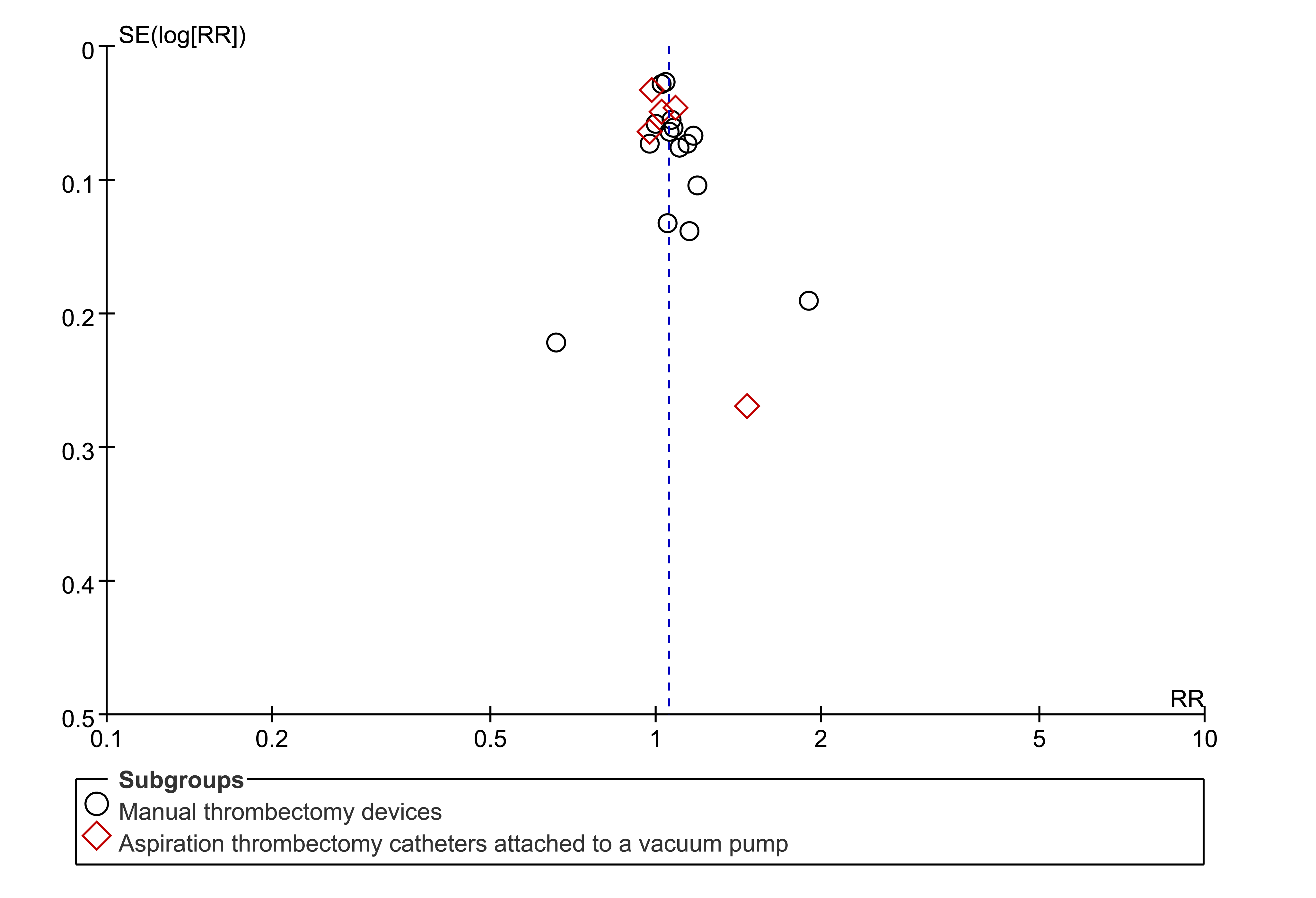

Supplement: Figure S5 — Funnel plot of the included studies in meta-analysis of post-procedure TIMI 3 flow. Footnote: The inverted and symmetrical funnel aspect can be observed for the assessed end points, with 95% of the studies lying within the confidence limit lines. This suggests that publication bias is not present among the included studies for the meta-analysis. (TIF) [file pone.0113481.s005.tif]
