# Supplementary material for: Adjunctive Manual Thrombus Aspiration during ST-Segment Elevation Myocardial Infarction: A Meta-Analysis of Randomized Controlled Trials
Source: PLoS One. 2014 Nov 18;9(11):e113481. doi: 10.1371/journal.pone.0113481 (PMC4236171; doi:10.1371/journal.pone.0113481)
Supplement: Checklist S1 — PRISMA checklist. (DOC) [file pone.0113481.s006.doc]

| **Section/topic** | **#** | **Checklist item** | **Reported on page #** |
| --- | --- | --- | --- |
| **TITLE** | | |  |
| Title | 1 | Adjunctive Manual Thrombus Aspiration during ST-Segment Elevation Myocardial Infarction: A Meta-analysis of  Randomized Controlled Trials | Title section |
| **ABSTRACT** | | |  |
| Structured summary | 2 | **Objective:** The aim of this study was to synthesize evidence by examining the effects of manual thrombus aspiration on clinical outcomes in patients with ST-segment elevation myocardial infarction (STEMI).  **Methods and Results:** A total of 26 randomized controlled trials (RCTs), enrolling 11,780 patients, with 5,869 patients randomized to manual thrombus aspiration and 5,911 patients randomized to conventional percutaneous coronary intervention (PCI), were included in the meta-analysis. Separate clinical outcome analyses were based on different follow-up periods. There were no statistically reductions in the incidences of mortality (risk ratio [RR], 0.86 [95% confidence interval [CI]: 0.73 to 1.02]), reinfarction (RR, 0.62 [CI, 0.31 to 1.32]) or target vessel revascularization (RR, 0.89 [CI, 0.75 to 1.05]) in the manual thrombus aspiration arm at 12 to 24 months of follow-up. The composite major adverse cardiac events (MACEs) outcomes were significantly lower in the manual thrombus aspiration arm over the long-term follow-up (RR, 0.76 [CI, 0.63 to 0.91]). A lower incidence of reinfarction was observed in the hospital to 30 days (RR, 0.59 [CI, 0.37 to 0.92]).  **Conclusion**: The present meta-analysis suggested that there was no evidence that using manual thrombus aspiration in patients with STEMI could provide distinct benefits in long-term clinical outcomes. | Abstract  section |
| **INTRODUCTION** | | |  |
| Rationale | 3 | Thrombus aspiration for ST-segment elevation myocardial infarction (STEMI) has been utilized for a long time and has received a level IIA endorsement according to the U.S. guidelines. In the recent years, there has been increasing interest in manual thrombectomy devices, and the evidence to date has suggested that manual thrombus aspiration, but not mechanical aspiration, is beneficial in reducing major adverse cardiac events (MACEs), including mortality, compared with conventional percutaneous coronary intervention (PCI) alone. In the largest randomized trial to date, the TASTE (Thrombus Aspiration in ST-Elevation myocardial infarction in Scandinavia) study suggested that routine manual thrombus aspiration before PCI provided no significant benefit to mortality over PCI alone in patients with STEMI at 30 days and 1 year of follow-up, settling the debate over the benefits of using manual thrombus aspiration in this setting. Recently, thrombectomy was downgraded in the ESC/EACTS revascularization guidelines from a class IIa level of evidence B recommendation to a class IIb level of evidence A recommendation. Despite two well-done updated meta-analyses recently performed on this topic by Kumbhani DJ et al, controversy exists regarding the combination of outcome effects over different follow-up durations. | Introduction section |
| Objectives | 4 | Because additional studies and prolonged follow-ups of earlier trials have now been reported, we performed an updated meta-analysis of the reperfusion markers of STEMI patients undergoing PCI with manual thrombus aspiration devices, and we performed separate analyses of clinical outcomes based on different follow-up periods | Introduction section |
| **METHODS** | | |  |
| Protocol and registration | 5 | Not applicable | - |
| Eligibility criteria | 6 | No restrictions were applied to the publication period of the articles. Only English-language studies were included. We selected studies in which patients with STEMI undergoing primary PCI or rescue PCI were randomly assigned either to manual thrombus aspiration followed by PCI or to PCI only. We only included studies that reported clinical outcome data and/or markers of post-procedure myocardial reperfusion. We excluded studies that performed thrombectomy only on saphenous vein grafts, studies that performed mechanical thrombectomy, studies of elective PCI and studies that compared one thrombectomy device to another. | Methods  “Data Sources and Searches” and “Study Selection” section |
| Information sources | 7 | We performed a computerized literature search of the PubMed, Web of Science, and Central databases for relevant articles published until September 2014. This search was supplemented with citation tracking of relevant review articles and prior meta-analyses. Furthermore, conference proceedings from the American College of Cardiology, American Heart Association, European Society of Cardiology, EuroPCR scientific sessions and Transcatheter Cardiovascular Therapeutics were scanned. | Methods  “Data Sources and Searches” section |
| Search | 8 | We performed a computerized literature search of the PubMed, Web of Science, and Central databases for relevant articles published until September 2014, using the Medical Subject Heading and keyword search terms myocardial infarction, ST-segment elevation myocardial infarction, STEMI, thrombus aspiration, thrombectomy, Diver, Pronto, Export, Thrombuster, Eliminate, Rescure, TVAC, revascularization, percutaneous coronary intervention, angioplasty and PCI. | Methods  “Data Sources and Searches” section |
| Study selection | 9 | We selected studies in which patients with STEMI undergoing primary PCI or rescue PCI were randomly assigned either to manual thrombus aspiration followed by PCI or to PCI only. We only included studies that reported clinical outcome data and/or markers of post-procedure myocardial reperfusion. We excluded studies that performed thrombectomy only on saphenous vein grafts, studies that performed mechanical thrombectomy, studies of elective PCI and studies that compared one thrombectomy device to another. | Methods  “Study Selection” section |
| Data collection process | 10 | The data were independently abstracted by two reviewers (Song-Bai Deng, Ling Wu). Agreement between the reviewers was evaluated by Kappa statistics. Disagreements were resolved through discussion, and a third reviewer (Qiang She) was involved to achieve a consensus when necessary. | Methods  “Data Extraction and Quality Assessment”  section |
| Data items | 11 | Not applicable |  |
| Risk of bias in individual studies | 12 | The bias of the included studies was assessed by the Cochrane group’s Cochrane Handbook for Systematic Reviews of Interventions. | Methods  “Data Extraction and Quality Assessment”  section |
| Summary measures | 13 | The primary clinical endpoint was all-cause mortality. The secondary endpoints were MACEs (composite of death, reinfarction, and target vessel revascularization), reinfarction, target vessel revascularization (TVR) and stent thrombosis. Angiographic and electrocardiographic outcomes that reflected post-procedure myocardial reperfusion included post-procedure myocardial blush grade (MBG) 3, thrombolysis and thrombin inhibition in myocardial infarction (TIMI) 3, and resolution of ST segment elevation (STR) > 70%. If manual and mechanical devices were both used in the same study, only data pertaining to manual aspiration thrombectomy were extracted. For all of the clinical outcomes, intention-to-treat analysis was utilized. The meta-analysis was performed using RevMan software, version 5.3 (Cochrane Collaboration). Summary risk ratios (RRs) and their corresponding 95% confidence intervals (CIs) were computed for each dichotomous outcome, using fixed or random effects models. | Methods  “Data Synthesis and Analysis” section |
| Synthesis of results | 14 | For outcomes with significant heterogeneity (Chi^2^ p<0.05 or I^2^>50%), the random effects model is reported in the text and figures; for all of the other outcomes, the fixed effects models are reported. The random effects models were employed for sensitivity analysis when the fixed effects models produced positive results. | Methods  “Data Synthesis and Analysis” section |

Page 1 of 2

| **Section/topic** | **#** | **Checklist item** | **Reported on page #** |
| --- | --- | --- | --- |
| Risk of bias across studies | 15 | We evaluated the level of evidence using the GRADE (Grades of Recommendation, Assessment, Development and Evaluation) approach. The GRADEpro software version 3.6 was used. We obtained our assessment by judging the designs of the studies, the risk of bias, inconsistency, and imprecision. | Methods  “Outcome Quality Assessment”  section |
| Additional analyses | 16 | We performed separate analyses of clinical outcomes based on different follow-up periods. The time frames were defined to reflect short-term (in hospital to 30 days), medium-term (6 to 9 months) and long-term (longer than or equal to 1 year) follow-ups, according to the different follow-up durations of the included studies. A subanalysis of the special thrombectomy devices (Rescue and TVAC) was performed. The random effects models were employed for sensitivity analysis when the fixed effects models produced positive results. | Methods  “Data Synthesis and Analysis” section |
| **RESULTS** | | |  |
| Study selection | 17 | The initial search obtained 641 potentially relevant publications. After reading the abstracts and the full texts, 26 RCTs were finally included, enrolling 11,780 patients, with 5,869 patients randomized to manual thrombus aspiration and 5,911 patients randomized to conventional PCI (Figure 1). | Paragraph 1 of “Results” section |
| Study characteristics | 18 | The follow-up periods varied between in-hospital and 5 years. Twelvetrials presented short-term follow-up results (in-hospital to 30 days), 8 trials presented medium-term follow-up results (6 to 8 months), and 2 trials presented long-term follow-up results (12 months). In addition, 5trials presented different follow-up periods: the TAPAS (The Thrombus Aspiration during Percutaneous Coronary Intervention in Acute Myocardial Infarction Study) and INFUSE-AMI (Infuse–Acute Myocardial Infarction; An Optical Frequency Domain Imaging Study) studies followed up from 30 days and then to up to 12 months, TASTE followed up from 30 days to 1 year [7,8], EXPIRA (The Thrombectomy With Export Catheter in Infarct-Related Artery During Primary Percutaneous Coronary Intervention Prospective, Randomized Trial) from 9 months and up to 24 months and VAMPIRE (VAcuuM asPIration thrombus Removal trial) from 1 month to 5 years.(Table 1) | Paragraph 1 of “Results” section  Table 1 |
| Risk of bias within studies | 19 | Among 24 included studies, most of them did not explicitly describe random sequence generation or allocation, and few presented attrition or reporting bias. Because the participants and personnel could not be blinded to the trials, the vast majority of studies were only blinded to outcome assessment. | Paragraph 1 of “Results” section  Figure S1 |
| Results of individual studies | 20 | See Figure2-6, Figure S2-4 | Figure2-6, Figure S2-4 |
| Synthesis of results | 21 | See Figure2-6, Figure S2-4 | Paragraph 2-7 of “Results” section  Figure2-6, Figure S2-4 |
| Risk of bias across studies | 22 | See Figure S1, Table 2 | See Figure S1, Table 2 |
| Additional analysis | 23 | See Figure2-5, Figure S2-4 | Results  Figure2-5, Figure S2-4 |
| **DISCUSSION** | | |  |
| Summary of evidence | 24 | This meta-analysis performed separate analyses based on different follow-up periods to compare the clinical outcomes of manual thrombus aspiration with those of conventional PCI. The main findings of this meta-analysis were that the use of manual thrombus aspiration devices could significantly reduce the incidence of short-term reinfarction and long-term MACEs, but it did not result in lower rates of death, reinfarcion or TVR over long-term follow-up despite improved post-procedure myocardial reperfusion. These results were driven mainly by the TASTE trial. | Paragraph 1 of “Discussion” section |
| Limitations | 25 | Our review had some limitations. First, this meta-analysis was not performed on individual patient data because complete data sets were not available. Second, only the TASTE trial was powered for mortality and the other clinical events reported, and the other included trials’ sample sizes were small. Third, the number of screened subjects or percentages of included vs. candidate subjects in a number of included studies were not accounted for, which is an important limitation that confounds how we should interpret our selection and outcome results. | Paragraph 5 of “Discussion” section |
| Conclusions | 26 | In summary, the present meta-analysis suggested that the use of manual thrombus aspiration devices could improve post-procedure myocardial reperfusion, but there was no evidence of a benefit in long-term clinical outcomes. | “Conclusion” section |
| **FUNDING** | | |  |
| Funding | 27 | No current funding sources for this study. | - |

*From:*  Moher D, Liberati A, Tetzlaff J, Altman DG, The PRISMA Group (2009). Preferred Reporting Items for Systematic Reviews and Meta-Analyses: The PRISMA Statement. PLoS Med 6(6): e1000097. doi:10.1371/journal.pmed1000097

For more information, visit: **www.prisma-statement.org**.

Page 2 of 2
